# Asynchronous calibration of quantitative computed tomography bone mineral density assessment for opportunistic osteoporosis screening: phantom-based validation and parameter influence evaluation

**DOI:** 10.1038/s41598-022-24546-2

**Published:** 2022-12-01

**Authors:** Stephan Skornitzke, Neha Vats, Taisiya Kopytova, Elizabeth Wai Yee Tong, Tobias Hofbauer, Tim Frederik Weber, Christoph Rehnitz, Oyunbileg von Stackelberg, Klaus Maier-Hein, Wolfram Stiller, Jürgen Biederer, Hans-Ulrich Kauczor, Claus-Peter Heußel, Mark Wielpütz, Viktoria Palm

**Affiliations:** 1 Clinic for Diagnostic and Interventional Radiology (DIR), Heidelberg University Hospital, Heidelberg, Germany; 2 Translational Lung Research Center (TLRC), German Center for Lung Research (DZL), University of Heidelberg, Heidelberg, Germany; 3 Division of Medical Image Computing, German Cancer Research Center, Heidelberg, Germany; 4 Pneumologie & Thoraxradiologie Weinheim, Weinheim, Germany; 5 Pattern Analysis and Learning Group, Department of Radiation Oncology, Heidelberg University Hospital, Heidelberg, Germany; 6 Diagnostic and Interventional Radiology With Nuclear Medicine, Thoraxklinik Heidelberg, Heidelberg, Germany; 7 Faculty of Medicine, University of Latvia, Riga, Latvia; 8 Faculty of Medicine, Christian-Albrechts-Universität zu Kiel, Kiel, Germany

**Keywords:** Metabolic bone disease, Osteoporosis, Metabolic bone disease, Osteoporosis, Diseases, Endocrinology, Diagnosis, Bone imaging, Health care, Medical imaging, Tomography, Computed tomography

## Abstract

Asynchronous calibration could allow opportunistic screening based on routine CT for early osteoporosis detection. In this phantom study, a bone mineral density (BMD) calibration phantom and multi-energy CT (MECT) phantom were imaged on eight different CT scanners with multiple tube voltages (80–150 kV_p_) and image reconstruction settings (e.g. soft/hard kernel). Reference values for asynchronous BMD estimation were calculated from the BMD-phantom and validated with six calcium composite inserts of the MECT-phantom with known ground truth. Relative errors/changes in estimated BMD were calculated and investigated for influence of tube voltage, CT scanner and reconstruction setting. Reference values for 282 acquisitions were determined, resulting in an average relative error between calculated BMD and ground truth of − 9.2% ± 14.0% with a strong correlation (*R*^2^ = 0.99; *p* < 0.0001). Tube voltage and CT scanner had a significant effect on calculated BMD (*p* < 0.0001), with relative differences in BMD of 3.8% ± 28.2% when adapting reference values for tube voltage, − 5.6% ± 9.2% for CT scanner and 0.2% ± 0.2% for reconstruction setting, respectively. Differences in BMD were small when using reference values from a different CT scanner of the same model (0.0% ± 1.4%). Asynchronous phantom-based calibration is feasible for opportunistic BMD assessment based on CT images with reference values adapted for tube voltage and CT scanner model.

## Introduction

Osteoporosis is defined by systemic bone mineral density (BMD) loss and microarchitectural deterioration, resulting in increased skeletal fragility and consequently a higher fracture risk^1^. Early detection of osteoporosis is of utmost importance, before an undetected progression of BMD-loss can lead to irreversible complications such as chronic pain, height loss with kyphosis causing posture impairment and eventually paraplegia resulting in lifelong physical disability^2,3^. Additionally, osteoporosis is a comorbidity of a multitude of medical conditions, e.g. chronic pulmonary disease, and osteoporosis itself has a negative effect on the outcome of other diseases. Osteoporosis affects about 500 million people and is one of the leading chronic diseases worldwide^4^. Pharmaceutic treatment of osteoporosis is limited in that it is not curative and may only prevent further fractures and disease progression. However, since the early stages of osteoporosis are silent, diagnosis is often only made when fractures have already occurred^5^. With current increases in overall life expectancy, osteoporosis can only be expected to increase in relevance in the future, as it is more prevalent in old age.

The reference standard to diagnose osteoporosis is dual-energy X-ray absorptiometry (DEXA)^6^. An alternative to DEXA are quantitative computed tomography (QCT) measurements, which allow for the detailed analysis of e.g. vertebrae and avoiding measurements of fractured bone, based on the three-dimensional nature of the data^7,8^. While these exams are justified for patients with a clinical suspicion of osteoporosis, opportunistic screening would allow to measure BMD in routine CT examinations. This could become a preventive approach without additional patient radiation exposure and without the need for specific screening programs^9^. Prospective BMD measurements with QCT rely on the use of a calibration phantom that is scanned simultaneously below the patient, whereas opportunistic screening attempts to estimate the BMD without the simultaneous use of calibration phantoms^9–11^. Some studies have previously investigated the potential for retrospective BMD measurements from routine CT. For example, Jang et al. published average CT numbers in the L1 vertebra obtained from more than 20,000 CT examinations, which can be used as a reference to identify patients with low BMD^12^. Nevertheless, this method does not allow exact BMD quantification and correlation to gold standard DEXA or QCT measurements. Another approach is to rely on asynchronous calibration measurements, where calibration data is obtained by intermittent QCT phantom scans, independently from the patient scans^13,14^. This approach allows for the retrospective assessment of BMD, as the calibration measurements can be performed and applied after the patient data have already been acquired. However, current studies on this topic are limited to specific acquisition protocols on specific CT scanners^13,14^. For widespread retrospective BMD assessment, calibration data is needed for each acquisition protocol on every CT scanner. Furthermore, studies have investigated the influence of some study parameters on BMD quantification, including the accuracy of retrospective BMD quantification depending on CT scanner, acquisition parameters and reconstruction settings. However, simultaneous changes of these parameters over a large parameter range have not been investigated in detail yet^11^. The aim of this phantom study was to evaluate the accuracy of BMD measurements with asynchronous phantom calibration for different CT scanners, acquisition parameters and reconstruction settings. Additionally, the influence of the CT scanners, acquisition parameters and reconstruction setting on the accuracy of the BMD measurements was assessed.

## Methods

No ethics approval was necessary for the phantom measurements in this study. The evaluation of patient data was approved by the institutional review board of the University Hospital Heidelberg under number S-937/2020 and performed in accordance with the Declaration of Helsinki 2013. Informed consent was waived and all methods were carried out in accordance with state-of-the-art guidelines and regulations.

### Data acquisition

#### Calibration phantom

**Figure 1:**
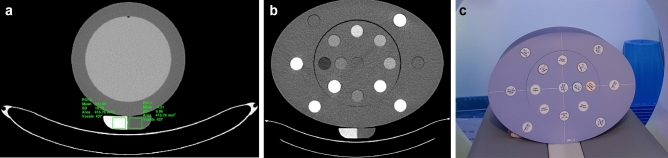
Image example from the calibration measurement (**A**) and the validation measurement with the MECT phantom (**B**, **C**). Images (A) and (**B**) were acquired on CT scanner #1 with a tube voltage of 120 kV_p_ and reconstructed with filtered-back projection using a soft tissue kernel (B30f.).

A commercially available QCT calibration phantom was used for asynchronous calibration measurements (OSTEO, Siemens Healthineers; Fig. 1a). This QCT calibration phantom consists of two phases corresponding to 0 mg/ml and 200 mg/ml hydroxylapatite^15^. The calibration phantom was scanned simultaneously with a commercially available image quality phantom (CATPhan 600, The Phantom Laboratory), which provided additional absorption (Fig. 1a).

#### Validation phantom

A commercially available multi-energy CT phantom (MECT Phantom, SunNuclear) with tissue-equivalent inserts was used for validation (Fig. 1b,c). Six inserts containing calcium composites were scanned. Ground truth equivalent hydroxylapatite concentrations were calculated based on the calcium content given by the manufacturer (Table 1). To this end, relative calcium content (Ca%) was multiplied with the density of the insert (ρ) to arrive at the calcium concentration in mg/ml. To further convert calcium concentrations to hydroxylapatite concentrations (BMD_equiv_), the calcium concentration of hydroxylapatite was calculated from its chemical formula: Ca_5_(PO_4_)_3_OH. Based on the molecular weights of the individual components of hydroxylapatite, 100 mg of hydroxylapatite contain 39.89 mg of Calcium (Ca). Inversely, we assume that an insert with a calcium concentration of 100 mg/ml is equivalent to a hydroxylapatite concentration of 251 mg/ml.

**Table 1:** Specifications of the phantom inserts. Calcium concentrations were calculated from given density and calcium fraction. Equivalent concentrations of Hydroxylapatite were calculated based on the calcium concentration according to the relative calcium content of Hydroxylapatite (i.e. 39.89%).

Insert	Nominal Density [g/cm^3^]	Calcium fraction [%]	Calcium concentration [mg/cm^3^]	Hydroxylapatit equivalent [mg/cm^3^]
Calcium, 50 mg/ml	1.17	4.36	50.09	127.65
Calcium, 100 mg/ml	1.24	8.18	101.80	255.07
Calcium, 300 mg/ml	1.55	19.76	305.90	766.74
CaCO3–30%	1.33	11.77	156.54	392.39
CaCO3–50%	1.56	19.62	306.07	767.21
HE Inner Bone	1.21	9.82	118.82	297.84

1 $$BM{D}_{equiv}=CA\%\cdot \rho \cdot 2.51$$

#### CT acquisition parameters

Eight CT scanners representing seven different scanner models from three manufacturers located at our institution and a nearby practice were included in the study (Table 2). For each scanner, all available settings for tube voltage were evaluated. Reconstruction settings were chosen to reflect the most commonly used clinical parameters on the respective scanner. These were intended to cover most of the potential patients’ data to be included in later evaluations. Both filtered-back projection and iterative reconstruction were included in the study with reconstruction kernels optimized for both soft-tissue and bone.

**Table 2:** Overview of included CT scanners and scan parameters.

Number	CT Scanner	Manufacturer	Tube voltages	Reconstruction kernels	Repetitions
1	SOMATOM Definition Flash	Siemens Healthineers	70, 80, 100, 120, 140	B30s, B40s, B70s, I30s, I40s, I70s	2
2	SOMATOM Force	Siemens Healthineers	70, 80, 90, 100, 110, 120, 130, 140, 150	Br36s, Br40s, Br69s, Br36s/3, Br40s/3, Br69s/3	2
3	Emotion 16	Siemens Healthineers	80, 110, 130	B20s, B30s, B60s	1
4	SOMATOM Definition AS	Siemens Healthineers	70, 80, 100, 120, 140	B30s, B40s, B70s, I30s, I40s, I70s	2
5	SOMATOM Definition AS	Siemens Healthineers	70, 80, 100, 120, 140	Br36, Br40, Br57, Bl36/3, Bl40/3, Bl57/3	1
6	IQon	Philips Healthcare	80, 100, 120, 140	YB, B, IMR1	1
7	Spectral CT 7500	Philips Healthcare	80, 100, 120, 140	YB, B, IMR1	2
8	Aquilion Lightning	Canon Medical Systems	80, 100, 120, 135	FC11, FC30, AiDR FC18, AiDR FC30	1

The following parameters were kept constant between all CT scanners for improved comparability: slice thickness 3 mm, slice increment 3 mm, rotation time 1 s, CTDI_vol_ 15 mGy. Data acquisition was repeated with a minimum gap of 6 months for four of the eight CT scanners to investigate changes in reference values over time, e.g. because of changes in scanner performance, scanner calibration or maintenance.

#### Reference values

For each acquisition, reference values were determined for the two phases of the calibration phantom by means of region of interest (ROI) evaluation. Rectangular ROIs with a size of 417 mm^2^ were placed in the calibration phantom for all slices covered by the image quality phantom (Fig. 1a). Images showing artifacts at the phantom edges in the z-direction were excluded from the measurement. A total of 64 images could be evaluated per measurement, corresponding to a length of 192 mm. Linear regression analysis was performed for each acquisition, determining the slope and intercept of a linear function to compute bone density or hydroxylapatite values from CT numbers:

2 $$BMD=Slope\cdot CTnumber+Intercept$$

### Evaluation

#### Validation of BMD quantification

The image data from the measurements of the validation phantom were analysed with a commercially available software tool from the phantom manufacturer (RapidCheck, SunNuclear), which automatically measures mean CT numbers of all inserts. CT numbers measured for inserts containing calcium composites were converted to bone-densities according to Eq. (2), using the previously obtained reference values. Calculated bone-densities were compared to the true equivalent hydroxylapatite concentrations of the insert (BMD_Truth_) as given in Table 1 (calculated with Eq. 1) to analyse measurement accuracy. Results are given in terms of relative error, e.g.

3 $$Re{l}_{Error}= \frac{BM{D}_{Measured}-BM{D}_{Truth}}{BM{D}_{Truth}}$$

Here, BMD_measured_ is the BMD calculated according to Eq. (2) using the reference values, while BMD_Truth_ is the ground truth calculated according to Eq. (1) (Table 1).

#### Influence of reference values

As a gold standard, a reference value has to be determined for each potential combination of CT scanner, tube voltage and reconstruction setting. In a multi-center study with different CT scanners and a diverse patient collective, where varying acquisition protocols and acquisition settings are used, the number of required reference measurements can be very high.

The validation data was analyzed to determine whether reference measurements are necessary for every potential combination of acquisition parameter, reconstruction setting and CT scanner. As described below, BMD was calculated using a reference value from a different acquisition and compared to the result using the actual reference value. If the difference in BMD is small, then it might not be necessary to have different reference measurements.

For example, CT-numbers from one acquisition, e.g. at 80 kV_p_ tube voltage, were converted to BMD (Eq. 2) by using reference values from a different acquisition, e.g. at 100 kV_p_ tube voltage. The calculated BMD was then compared to the ground truth (Eq. 3) and to the BMD calculated with the actual reference value, i.e.:

4 $${Diff}_{100kV vs 80kV }=\frac{BM{D}_{100kV vs 80kV}-BM{D}_{80kV}}{BM{D}_{80kV}}$$

Here, BMD_80kV_ is the BMD calculated with the actual reference value at 80 kV_p_. BMD_100kV vs 80 kV_ is calculated from the CT-numbers acquired at 80 kV_p_ tube voltage, but converted to BMD with the reference values (i.e. slope and intercept; see Eq. 2) measured with a tube voltage of 100 kV_p_ instead. For each scanner, every potential combination of tube voltage was considered. The analysis was performed separately for the following factors (consider also Table 2):

1. CT Scanner
2. Tube voltage
3. Reconstruction setting
4. Repeated measurements (> 6-months time difference)

Regarding the comparison of CT scanners, data were analyzed while considering all scanners, considering only same-manufacturer scanners and considering only same-model scanners. Data were matched between CT scanners with exact matches for tube voltage, but reconstruction kernels were regarded as interchangeable in the following three groups:

1. Filtered back-projection with soft-tissue kernel (e.g. “B” or “B30f.”)
2. Filtered back-projection with hard kernel (e.g. “YB” or “B70f.”)
3. Iterative reconstruction (e.g. “IMR” or “I30f.”)

#### Statistical analysis

A linear correlation coefficient (Pearson’s r) was calculated to assess the correlation between calculated bone density values and ground truth.

To determine which factors influence BMD quantification, two general linear models were trained to perform analysis of covariances for both the calculated BMD (Eq. 2) as well as the relative error compared to the ground truth (Eq. 3). The following independent variables were considered for both general linear models: tube voltage, CT scanner, reconstruction setting, evaluated insert, repetition. All interactions between the independent variables were included in the model. Values from all six inserts were considered simultaneously by considering the type of insert as an independent variable. Because of the differences in material composition between the different inserts (Table 1), the evaluated insert was modeled as a categorical variable. Both models were built in a multi-step design, removing non-significant effects until all variables left had a significant effect or were part of a significant interaction effect.

Further analysis was done to determine which factors have to be considered when obtaining reference values. Analysis of variances (ANOVA) was performed to compare relative errors of BMD quantification (Eq. 3) when varying reference values. Relative errors in determined BMD when varying reference values for different influencing factors (e.g. tube voltage, etc.) were compared by Tukey’s test to the relative error for the gold standard BMD quantification, where the actual reference value was used. Additionally, differences in calculated BMD values (Eq. 4) where tested for non-zero differences with a one-tail Student’s *t*-test.

A *p*-value of 0.05 was chosen as the threshold for statistical significance. The analysis was performed using MATLAB 2021a (Mathworks) and SAS 9.4 (SAS Institute).

#### Experimental application

To experimentally explore the application of the determined reference values for BMD quantification, data from a single patient was retrospectively evaluated. The patient was acquired on CT Scanner #4 with two different acquisition protocols. Acquisition #1 was performed at 100 kV_p_ tube voltage with a tube current–time product of 21 mAs and reconstructed using the I40s Kernel with a slice thickness of 0.7 mm. Acquisition #2 was performed together with the calibration phantom at 80 kV_p_ tube voltage with a tube current–time product of 120 mAs, and reconstructed using the S80s Kernel with a slice thickness of 10 mm. BMD was calculated with the determined reference values from acquisition #1. BMD was measured with a commercial application for BMD quantification (syngo Osteo CT; Siemens Healthineers) for acquisition #2.

## Results

A total of 473 CT acquisitions were performed, including 282 acquisitions for reference measurements and 191 acquisitions for validation. These acquisitions cover a parameter space of eight CT scanners, seven CT scanner models, three manufacturers, ten tube voltages, 22 image reconstructions, and up to two repetitions.

### Validation of BMD quantification

**Figure 2:**
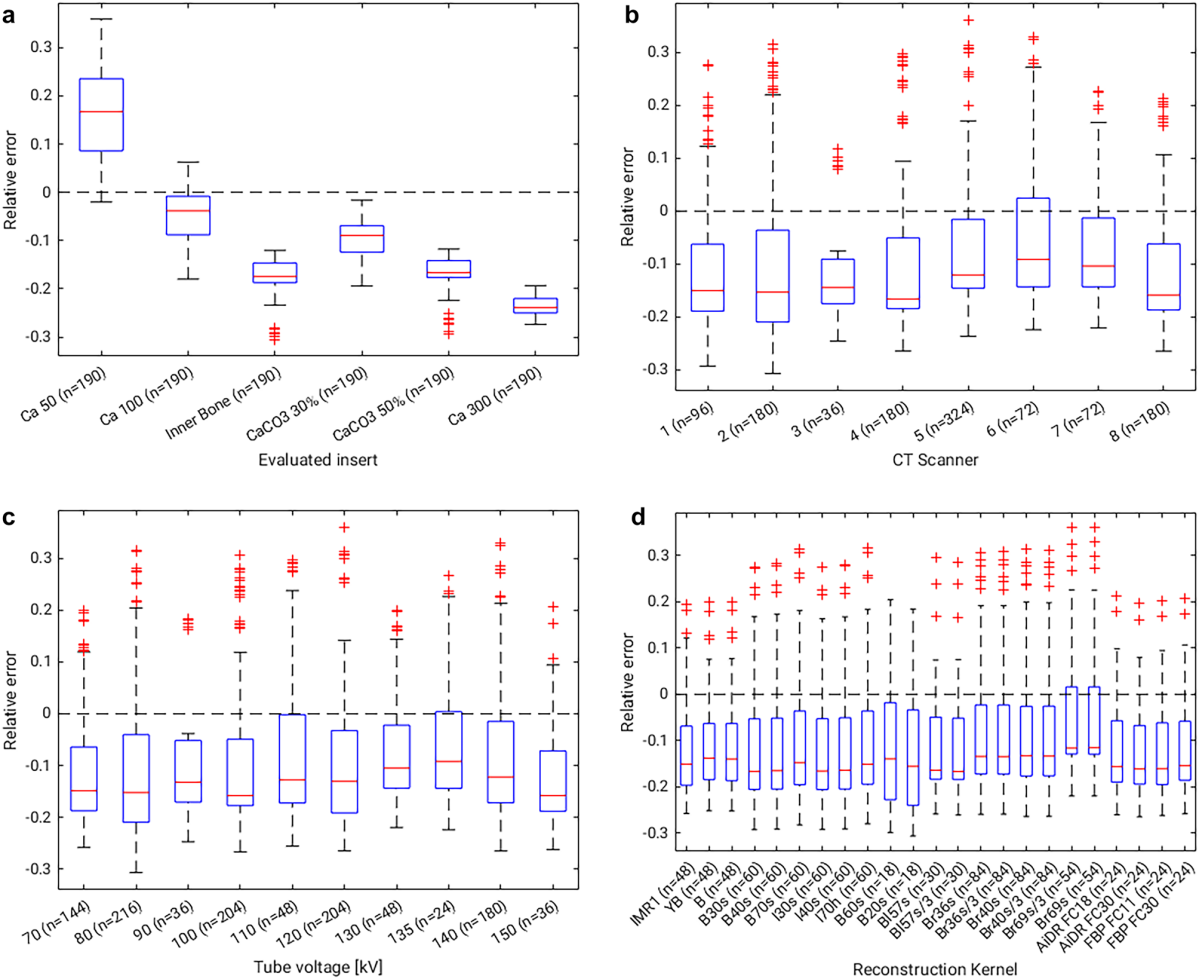
Relative error of computed bone mineral density compared to the calculated ground truth values over the different investigated parameters: (**A**) phantom insert (**B**) CT scanner (**C**) tube voltage (D) reconstruction setting. Note the differences in sample sizes for the different parameters (also Table 2).

Comparing the determined BMD values for the different inserts to the ground truth showed an average relative error of − 9.2% ± 14.0%. Correlation between calculated bone density values and ground truth was very strong with *R*^2^ = 0.99 (*p* < 0.0001). Considering inserts labelled as “Calcium” only (i.e. 50, 100, and 300 mg calcium; Table 1), the average relative error was − 2.0% ± 15.4%. Differences in relative error could be observed between the inserts, as shown in Fig. 2a. Further differences in relative error could be observed between CT scanners and tube voltages (Figs. 2b,c). On average, absolute values of relative error decreased with increasing tube voltage (Fig. 2c). The differences in relative error between different reconstruction kernels were small (Fig. 2d).

Statistical models for BMD quantification showed a good fit and a significant influence of independent variables on both the absolute BMD values (*p* < 0.0001; *R*^2^ = 0.99) and the relative error to ground truth (*p* < 0.0001; *R*^2^ = 0.99). While tube voltage, CT scanner and evaluated insert had a significant effect in both models, the repetition was only significant as part of interaction effects. The reconstruction setting was not a significant effect in either of the models. Consequently, only tube voltage, CT scanner and ground truth BMD can be considered to have a significant influence on BMD quantification. Reported p-values for all effects included in the final model are shown in Table 3.

**Table 3:** Results of statistical analysis from the two general linear models for the calculated bone density and the relative error, showing the F- and p-values for the remaining effects. Interaction effects are indicated by an asterisk between the individual effects. Note that non-significant effects can remain part of the model if they appear in significant interaction effects (i.e. Repetition).

Independent effect(s)	F-value	*p*-value
**Calculated bone density**		
Tube voltage	168.23	< .0001
CT scanner	35.61	< .0001
Tube voltage*CT scanner	23.32	< .0001
Insert	2796.79	< .0001
Tube voltage*Insert	48.74	< .0001
CT scanner*Insert	15.67	< .0001
Tube voltage*CT scanner*Insert	10.66	< .0001
Repetition	3.76	0.0528
Tube voltage*Repetition	2.83	0.0928
Repetition*CT scanner	10.35	< .0001
Tube voltage*Repetition*CT scanner	7.41	< .0001
**Relative error**		
Tube voltage	458.8	< .0001
CT scanner	45.49	< .0001
Tube voltage*CT scanner	30.65	< .0001
Insert	20.1	< .0001
Tube voltage*Insert	801.83	< .0001
CT scanner*Insert	34.92	< .0001
Repetition	2.82	0.0931
Tube voltage*Repetition	1.82	0.1772
Repetition*CT scanner	8.7	< .0001
Tube voltage*Repetition*CT scanner	5.78	0.0006

### Influence of reference values

**Figure 3:**
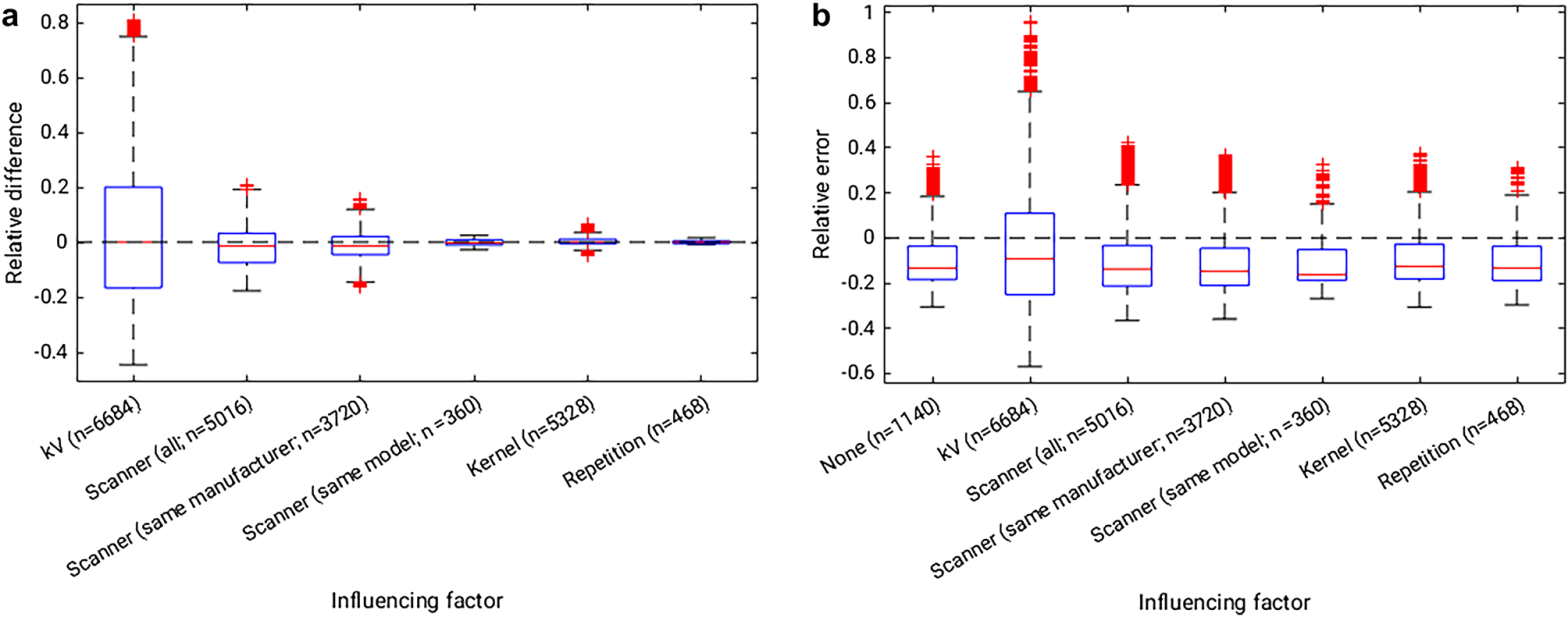
(**A**) Relative difference between calculated bone mineral density values when varying the reference value used for the calculation. “Scanner (all)” refers to using matching reference values (i.e. same tube voltage and similar reconstruction kernel) from any of the other scanners. (**B**) Relative error compared to ground truth when varying the reference value used for the calculation, compared to the value calculated when using the actual reference value (Influencing factor: “None”).

Comparing the results achieved when using reference values from a different acquisition to the results achieved with the actual reference values (Eq. 4) shows that the tube voltage (average relative difference: 3.8% ± 28.2%) and CT scanner (− 5.6% ± 9.2%) have a strong influence on the results (Fig. 3a). Using reference values from a different CT scanner of the same scanner model showed little impact on the results (0.0% ± 1.4%). Differences were larger when using reference values from any CT scanner of the same manufacturer (− 5.5% ± 6.6%) and largest when using any other CT scanner, regardless of manufacturer (− 5.6% ± 9.2%). The influence of the reconstruction settings was comparatively low (0.2% ± 0.2%), with a mean difference of 0.0% ± 0.1% when comparing iterative reconstruction to filtered back-projection. Comparisons between repeated calibration measurements had a mean difference of 0.1% ± 0.7%. A significant non-zero difference in calculated BMD was found for tube voltage, CT scanners (all evaluations) and reconstruction setting, but not for the repetition (*p* < 0.05).

Similarly, analyzing the relative error between calculated BMD and ground truth when using reference values from different acquisitions shows error levels on par with the gold standard when varying repeated measurements (− 9.5% ± 13.9%), reconstruction settings (− 8.7% ± 14.3%), or when using a different CT scanner of the same scanner model (− 10.5% ± 14.2%; Fig. 3b). Absolute values of relative errors are slightly larger comparing CT scanners of the same manufacturer (− 11.3% ± 14.2%), or any other CT scanner regardless of manufacturer (− 11.0% ± 14.7%), or using reference values acquired for a different tube voltage (− 5.9% ± 26.5%).

For the analysis of the relative error depending on varying reference values, the ANOVA reported a significant difference in relative errors between the groups (*p* < 0.0001). Pair-wise comparison to the relative error of the gold standard BMD quantification using the actual reference value showed significant differences for tube voltage and CT scanners from the same manufacturer (*p* < 0.05). No significant differences were found for the reconstruction settings, repetition, CT scanners from all manufacturers or CT scanners of the same model.

**Figure 4:**
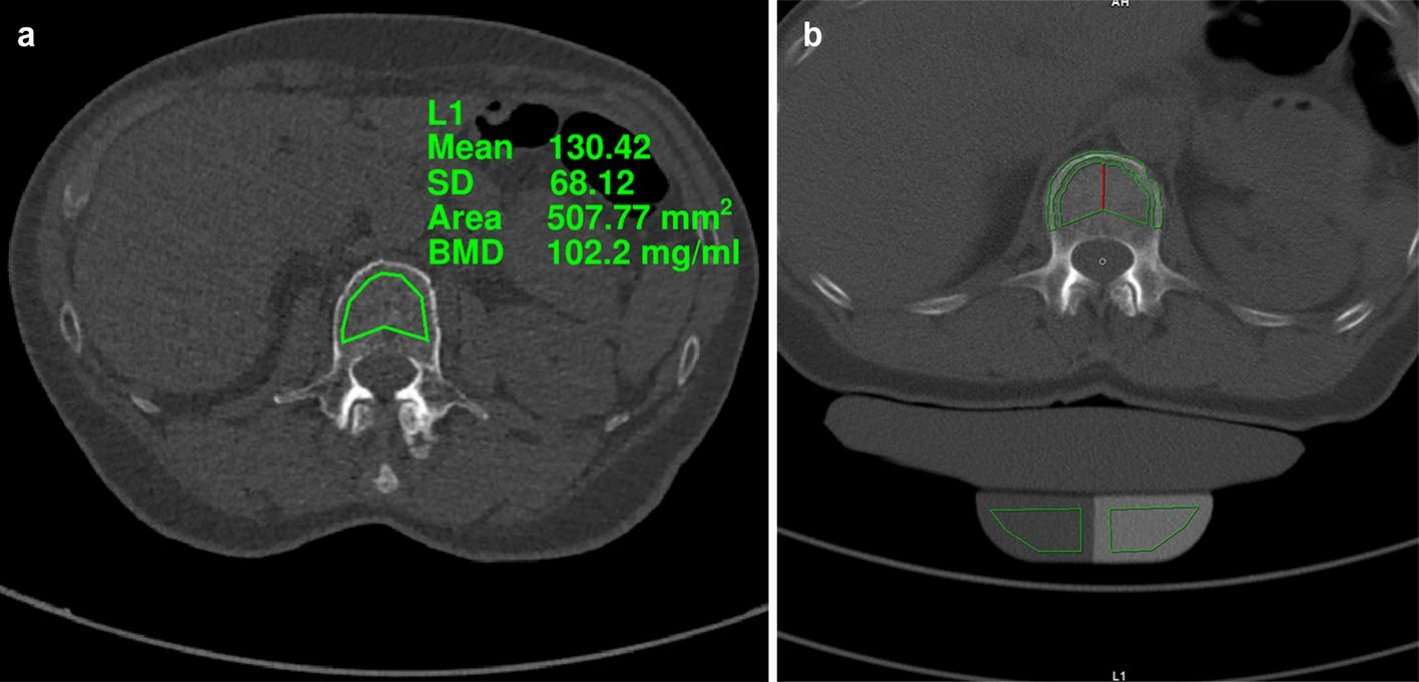
Image example comparing bone mineral density (BMD) measurements in terms of hydroxylapatit concentrations with synchronous and asynchronous calibration. (**A**) BMD measurement in the first vertebra of the lumbar spine (L1) of an 84-year-old female, showing a BMD of 102.2 mg/ml. BMD is calculated based on CT numbers of a non-contrast CT scan using the asynchronous reference values determined in this study. (**B**) Automated segmentation of L1 in the same 84-year-old female scanned with the reference phantom using syngo Osteo CT (Siemens Healthineers). Reported BMD of 96.2 mg/ml for L1 using synchronous calibration.

Experimental application of the asynchronous calibration for opportunistic BMD measurements in a patient (Fig. 4a) showed BMD values in line with those obtained from a commercially available solution using synchronous calibration (syngo Osteo CT, Siemens Healthineers; Fig. 4b).

## Discussion

With this study we showed that opportunistic screening of BMD is potentially feasible by using post-hoc generated, asynchronous phantom calibration for multiple CT scanners with a wide range of acquisition settings. Additionally, the influence of individual factors on the accuracy of BMD quantification was determined in this study, showing which factors should be considered for reference measurements. The results show that the acquired reference values can be used to measure BMD with an acceptable accuracy for screening purposes in a wide variety of acquisition and reconstruction settings. The results regarding the influencing factors could be used for further improvement of CT BMD measurements. While DEXA can also be influenced by factors like patient size and fat distribution^16^, DEXA or QCT is still warranted for follow-up to obtain a more accurate determination of individual risk for osteoporosis.

Some previous studies reported more accurate results for asynchronously calibrated BMD measurements than this study, but these studies only investigated limited acquisition and reconstruction settings over a smaller range. For example, acquisitions between 70 and 150 kV_p_ were included in the current study, where the error was larger for small tube voltages (see Fig. 2c). While Garner et al. also analyzed tube voltages of 80 kV_p_, 100 kV_p_ and 140 kV_p_, they focused on CT numbers instead of bone density^17^. In comparison, Wang et al. reported a consistent underestimation of bone density by 1.4–6.7% in their phantom study, but only investigated scans at a tube voltage of 120 kV_p_^14^. Michalski et al. also reported a 2.8% mean percent difference between asynchronous BMD measurements and regular phantom-based QCT for human cadaver scans at 120 kV_p_^18^. Woisetschläger et al. reported an average underestimation of 8–14% when comparing asynchronous measurements to those using internal CT calibration in human patients^19^. Despite the fact that the current study compares asynchronous measurements to synchronous phantom calibration instead of internal calibration, the results are similar. Generally, results for BMD measurements after asynchronous calibration are considered slightly less accurate than those with simultaneous calibration^11^. Furthermore, analysis of factors influencing the measurement accuracy shows that tube voltage has a strong influence on results, while other factors like image reconstruction and changes over time play a limited role only. In consequence, CT scans should be performed at the same tube voltage as the asynchronous calibration, or calibration has to be performed for all available tube voltages. These results are in line with those reported in a literature review by Brunnquell et al., who also cited a strong influence of tube voltage^11^. Furthermore, similar results were reported by Garner et al., who analyzed the influence of the tube voltage on CT numbers measured in L1 trabecular bone^17^. Additionally, the effect of using calibration data from a different CT scanner was investigated in this study. The results suggest that calibration data from one CT scanner might be used on different CT scanners of the same model with a moderate error, but that data from different CT scanner models should not be considered interchangeable. Even though differences between CT scanners were slightly larger when comparing different manufacturers, the difference to the ground truth was only statistically significant for CT scanners of the same manufacturer. This might be explained by the overall low number of 8 included CT scanners or the unbalanced design of the study, where most CT scanners came from a single manufacturer while also exhibiting the most technical inter-scanner variation. Consequently, further analysis seems necessary. Furthermore, specific calibration for different image reconstruction settings might not be strictly necessary, as the observed differences were small.

One limitation of this study is that a generalized MECT phantom was used for validation instead of specialized BMD phantoms like the European spine phantom^20^. The available inserts for the MECT phantom contain relatively high calcium concentrations, surpassing what would be observed during regular BMD measurements in clinical practice and potentially leading to photon starvation artifacts at low tube voltage. Yoganandan et al. reported an average BMD of 169.7 mg/cm^3^ for L3 and a maximum of 429.9 mg/cm^3^ over the cervical, thoracic and lumbar vertebrae in healthy males, while the maximum considered in this study was 767.21 mg/cm^3^ (Table 1)^21^. Furthermore, results show differences in relative measurement errors depending on the insert, which might be based on the differences in chemical composition of the inserts. As the evaluated phantoms use artificial reconstructions of the investigated tissues, the spectral X-ray absorption of the inserts might be most accurate for acquisitions at standard settings, i.e. 120 kV_p_. The wide range of tube voltages from 70 kV_p_ up to 150 kV_p_ considered in this study might exacerbate any inaccuracies. The results show that the relative error is reduced when considering only the phantom inserts labeled as “calcium”.

While a large number of CT scanners from three different manufacturers were included in this study, not all manufacturers could be included and results may differ for the CT scanners not investigated in this study. Additionally, only a limited number of factors was considered in this study. For example, differences in CTDI_vol_ were not investigated, only up to one repeated measurement was performed, the patient size was not considered, and changes based on scanner calibration or maintenance were not evaluated. Only a limited number of reconstruction kernels were evaluated and quantitative kernels for post-processing available on some CT scanners were not included in the study. However, the evaluated kernels were chosen to closely match clinical practice and the results indicate that the reconstruction kernel has limited influence on the BMD quantification. Moreover, different phantom sizes were used for calibration and for validation, which might limit measurement accuracy.

Furthermore, with the increased availability of Dual-Energy CT (DECT), spectral methods for bone mineral quantification have been developed as well and should be investigated^22^. In the future, further validation of the technique is planned by correlating results from BMD measurements with asynchronous phantom calibration to DEXA measurements in a large patient collective.

In conclusion, the results show that opportunistic calculation of BMD from CT images using asynchronous phantom calibration is possible with a relative error of about − 9.2%. The main factors that have to be considered for calibration are the tube voltage and the scanner model.

## Data Availability

The datasets generated during and/or analysed during the current study are available from the corresponding author on reasonable request.
